# Transfusion-transmissible viral infections among blood donors at the North Gondar district blood bank, northwest Ethiopia: A three year retrospective study

**DOI:** 10.1371/journal.pone.0180416

**Published:** 2017-07-05

**Authors:** Belete Biadgo, Elias Shiferaw, Berhanu Woldu, Kefyalew Addis Alene, Mulugeta Melku

**Affiliations:** 1 Department of Clinical Chemistry, School of Biomedical and Laboratory Sciences, College of Medicine and Health Sciences, University of Gondar, Gondar, Ethiopia; 2 Department of Hematology and Immunohematology, School of Biomedical and Laboratory Sciences, College of Medicine and Health Sciences, University of Gondar, Gondar, Ethiopia; 3 Department of Epidemiology and Biostatistics, Institute of public Health, College of Medicine and Health Sciences, University of Gondar, Gondar, Ethiopia; University of Cincinnati College of Medicine, UNITED STATES

## Abstract

**Background:**

Transfusion-transmissible viral infections, such as hepatitis C virus (HCV), hepatitis B virus (HBV), and human immunodeficiency virus (HIV), remain a major public health problem in developing countries. The prevalence of these viral infections among blood donors may reflect the burden of these diseases among populations. Therefore, the aim of this study was to assess the sero-prevalence of transfusion-transmissible viral infections among blood donors.

**Methods:**

A retrospective study was conducted using data obtained from registration books of blood donors from the Ethiopian North Gondar District Blood Bank from 2010 to 2012. Descriptive statistics, such as percentages, medians and interquartile ranges were computed. A binary logistic regression model was fitted to identify factors associated with each viral infection. The odds ratio with a 99% confidence interval was calculated. A p-value < 0.01 was considered statistically significant.

**Result:**

A total of 6,471 blood donors were included in the study. Of these, 5,311 (82.1%) were male, and 382 (5.9%) were voluntary blood donors. Overall, 424 (6.55%) of the blood donors were sero-reactive for at least one transfusion-transmissible viral infection. Of all study participants, 233 (3.6%) were sero-reactive for HBV, 145 (2.24%) were sero-reactive for HIV, and 51 (0.8%) were sero-reactive for HCV. Four (0.062%) of the study’s participants were co-infected: 3 (75%) with HBV-HCV and 1 (25%) with HIV-HBV-HCV. Being a farmer, unemployed or employed donor was significantly associated with transfusion-transmissible viral infections compared to being a student donor.

**Conclusion:**

The prevalence of transfusion-transmissible viral infections is substantial and has increased overtime. Hence, it demands more vigilance in routine screening of donated blood prior to transfusion. Further community-based studies to identify societal risk factors are necessary.

## Introduction

Blood transfusion saves millions of lives worldwide each year [1]. However, transfusion-transmissible infections (TTIs) are a major problem associated with blood transfusion, particularly in developing countries [1]. The magnitude of this problem is directly related to the prevalence of TTIs among blood donors [2]. In several settings, human immunodeficiency virus (HIV), hepatitis B virus (HBV), and hepatitis C virus (HCV) are the major TTIs [3–5]. Each blood transfusion carries a risk of transmitting blood-borne pathogens [6]. For instance, in sub-Saharan Africa, 5–12% of patients who received blood transfusions are at risk of post-transfusion hepatitis and HIV infections [7]. For this reason, the prevention and control of TTIs is the leading concern and priority of the World Health Organization (WHO) and blood transfusion programs in Sub-Saharan African countries, including Ethiopia [8].

TTIs can pose risks for healthcare workers. Because the prevalence of transfusion-transmissible pathogens is high in the general population, the occupational risk of exposure to these pathogens for healthcare workers poses a major public health challenge. According to WHO estimates, 3 million healthcare workers experience percutaneous exposures to blood pathogens worldwide each year. Of these, 2 million are exposed to HBV [9]. A study conducted in Bulle Hora, Ethiopia, showed that HBV was higher among healthcare workers (7.3%) than non-healthcare workers (0.9%) [10].

The prevalence of TTIs among blood donors varies across the world, as well as within countries, contingent on the prevalence of these viruses in the general population [11,12]. According to a WHO report, Ethiopia is classified under geographical regions with intermediate to hyper-endemic viral hepatitis infections [13]. The prevalence of viral hepatitis varies within districts and target groups in Ethiopia [14–23]. The overall pooled prevalence of HBV was 7.4%. In sub-groups, the prevalence varied at 5.2% in HIV infected individuals, 8.0% in community based studies, 8.4% in blood donors, 11.0% in immigrants, and 6.9% in other groups [24]. Similarly, the prevalence of HCV and HIV is common in Ethiopia with regional differences. A study conducted among clinically suspected cases in Gondar indicated a high prevalence of HCV at 12.4% [14].

Several programs have been implanted in Ethiopia to reduce the burden of HBV in the community. These programs include the following: the Expanded Programme on Immunization Policy, updated in 2007 and including childhood immunization against HBV using a pentavalent form at ages 6, 10 and 14 weeks after birth; implementing antenatal screening for HBsAg of all pregnant women and the vaccination of their babies at birth; and recommending the vaccination of high risk groups such as health professionals against HBV. However, these programs have not been routinely enforced in most healthcare settings across the country[25]. A study conducted among healthcare workers in Bahir Dar, northwest Ethiopia, reported that HBV vaccination status was low. Only 5.4% reported that they took three or more doses of the HBV vaccine [26]. Similarly, another study revealed that the status of the HBV vaccination among surgeons practicing in Ethiopia was low, with only 24 out of 98 surgeons (23.5%) having received the vaccination [27].

Quality-guaranteed screening of all donated blood for TTIs, including HIV, HBV, and HCV, is recommended by the WHO and adopted by the Ethiopian government for the provision of safe and efficacious blood and blood components [28]. This includes the selection of eligible blood donors, the collection of blood, the processing and testing of the donated blood, the issuing of compatible blood, and safe administration of the blood to recipients. In response to this strategy, Ethiopia revoked responsibilities for blood transfusion from the Ethiopian Red Cross Society and granted it to the National Blood Transfusion Service Agency, a government agency managed under the Federal Ministry of Health and Regional Health bureau created in 2010 to ensure blood safety and accessibility [29]. Twenty-five blood banks were functional in the country in 2014. The North Gondar District Blood Bank is one of the blood banks located in northwest Ethiopia [29].

Monitoring time trends in TTIs among blood donors provides evidence to assess the effectiveness of blood supply screening programs, and may indicate changes in disease prevalence in communities. There are few epidemiological studies conducted in Ethiopia on TTIs among blood donors [16–18,20,21]. However, data are limited between 2010 and 2012 in northwest Ethiopia for the analysis of trends. Therefore, the aim of this study was to assess the sero-prevalence of HBV, HCV, and HIV among blood donors at the North Gondar District Blood Bank, northwest Ethiopia, between 2010 and 2012.

## Materials and methods

### Study design and setting

A retrospective cohort study was conducted at the North Gondar District Blood Bank among blood donors who donated blood from 2010 to 2012. The Blood Bank is in Gondar town, located 738 km northwest of the capital city, Addis Ababa. It provides services for a catchment population of approximately five million people in North Gondar and neighboring districts. On average, the blood bank collects 2,500 units annually. The majority of this blood is used for emergency, surgical, and gynecological cases.

### Study population

Study participants were all blood donors who donated blood at the North Gondar District Blood Bank from 2010 to 2012. They consisted of voluntary and replacement (family) blood donors who weighed more than 50 kg and were older than 17 years of age. A total of 6,471 blood donor records were reviewed and included in the study.

### Serological investigations

Serum or plasma samples were tested for HBV, HIV and HCV using the Enzyme Linked Immunosorbent Assay (ELISA) (HIV1/2: Vironostika HIV Uni-Form II Ag/Ab fourth generation ELISA, Bio-Merieux, Boxtel, Netherlands; HBsAg: a third generation ELISA, Hepanostika HBsAg UNi-Form II, Bio-Merieux, Boxtel, Netherlands; HCV: Human anti-HCV third generation ELISA, HumanGasellschaft for Bio-chemical and diagnostic MbH, Germany). All tests were performed according to the manufacturer’s instructions.

### Data collection and statistical analysis

Data on socio-demographic variables and laboratory test results were collected from blood donors’ registration books using the data extraction format. Collected data were then cross-checked for completeness, entered into Epi Info software (version 3.5.1), and then transferred to SPSS version 20 software for analysis. Descriptive statistics were performed, and the results were presented in tables. Both bivariate and multivariable binary logistic regression models were fitted to identify factors associated with each viral infection. The odds ratio and its 99% confidence interval were used to determine the strength of the association. A p-value <0.01 in the multivariable binary logistic regression analysis was considered to be statistically significant.

### Ethical considerations

The study was conducted after ethical clearance was obtained from the School of Biomedical and Laboratory Sciences, College of Medicine and Health Sciences, the University of Gondar. Permission was also obtained from the head of North Gondar District Blood Bank. Data were kept in a confidential manner. As the study used secondary data, informed consent was not sought from study participants.

## Results

### Demographic characteristics

A total of 6,471 individuals donated blood and were screened for viral infections during the three year period at the Gondar District Blood Bank. Approximately 5,311 (82.1%) and 3,070 (47.4%) of the blood donors were males and aged 18–25 years, respectively. The median age of the participants was 26 years (range: 48 years). Almost all of the 6,089 (94.1%) blood donations were obtained from a replacement donation (Table 1).

**Table 1:** Demographic characteristics of blood donors at the North Gondar District Blood Bank, northwest Ethiopia, 2010 to 2012.

Variables	Categories	Frequency	Percent
**Age (years)**	18–25	3,070	47.4
	26–35	2,191	33.9
	36–45	846	13.1
	>45	364	5.6
**Sex**	Male	5,311	82.1
	Female	1,160	17.9
**Occupation**	Student	1,842	28.5
	Farmer	1,728	26.7
	Government employed	947	14.6
	Private employed	296	4.6
	Self-employed	1,068	16.5
	Unemployed	590	9.1
**Type of donation**			
	Voluntary	382	5.9
	Replacement	6,089	94.1

### Transfusion-transmissible viral infection prevalence

Overall, 424 (6.55%; 99% CI: 5.76%, 7.34%) blood donors had serological evidence for at least one TTI (i.e., HBV, HCV, or HIV). Four (0.062%) blood donors were co-infected with more than one viral infection: 3(75%) with HBV-HCV and 1(25%) with HIV-HBV-HCV infections. The number of donors gradually increased from 2,006 (31.0%) in 2010 to 2,294 (35.4%) in 2012. The overall prevalence of TTIs also increased from 100 (5.0%) in 2010 to 213 (9.28%) in 2012 (Table 2).

**Table 2:** Frequency of HIV, HBV and HCV infections with respect to donation year among blood donors at the North Gondar district Blood Bank, northwest Ethiopia.

Year of donation	Total screened N (%)	HBV positive N (%)	HCV positive N (%)	HIV positive N (%)	Total sero- prevalence (HBV, HCV, or HIV) N (%)
2010	2,006 (31.0%)	57 (2.8%)	12 (0.6%)	36 (1.8%)	100 (5.0%)
2011	2,171(33.5%)	55 (2.5%)	10 (0.5%)	46 (2.1%)	111 (5.11%)
2012	2,294 (35.4%)	121 (5.3%)	29 (1.3%)	63 (2.7%)	213 (9.28%)
Total	6,471(100%)	233 (3.6%)	51 (0.8%)	145 (2.24%)	424 (6.55%)

### Sero-prevalence and associated factors of HBV infection

The overall sero-prevalence of HBV was 233 (3.6%; 99%CI: 3.0%, 4.2%) and was 4.14% among males and 3.48% among females. In the multivariable binary logistic regression analysis, farmer (AOR = 2.20; 99%CI: 1.25, 3.89), employed (AOR = 2.48; 99%CI: 1.46, 4.23), and unemployed (AOR = 4.00; 99%CI: 2.15, 7.46) donors were at a higher risk of HBV infection compared to student donors. In addition, the odds of HBV among people who donated blood in 2012 were almost two times as likely of having HBV among people who donated blood in 2010 (AOR = 1.84; 99%CI: 1.20, 2.80) (Table 3).

**Table 3:** Sero-prevalence of HBV infection according to socio-demographic characteristics of blood donors at the North Gondar District Blood Bank, northwest Ethiopia.

Variables	HBV		COR (99%CI)	AOR (99%CI)
	Reactive	Non-reactive		
**Age group**				
18–25	103	2,967	1.00	
26–35	75	2,116	1.02(0.69, 1.52)	
36–45	37	809	1.32(0.8, 2.2)	
>45	18	346	1.49(0.76, 2.2)	
**Sex**				
Male	185	5,126	1.00	
Female	48	1,112	1.2(0.78, 1.8)	
**Occupation**				
Student	32	1,810	1.00	1.00
Farmer	63	1,665	**2.14(1.24, 3.77)**	**2.20(1.25, 3.89)**
Employed*	97	2,214	**2.49(1.45, 4.21)**	**2.48(1.46, 4.23)**
Unemployed	41	549	**4.22(2.3, 7.9)**	**4.00(2.15, 7.46)**
**Type of donation**				
Voluntary	5	377	1.00	
Replacement	228	5,861	2.93(0.91, 9.74)	
**Year of donation**				
2010	57	1,949	1.00	1.00
2011	55	2,116	0.89 (0.54, 1.45)	0.87 (0.53, 1.43)
2012	121	2,173	**1.90 (1.23, 2.90)**	**1.84 (1.20, 2.80)**

COR: Crude Odds Ratio; AOR: Adjusted Odds Ratio; CI: Confidence Interval; and

*Employed: government employed, private employed and self-employed

### Sero-prevalence and associated factors of HCV infection

The sero-prevalence of HCV infection among blood donors was 51 (0.80%; 99%CI: 0.5%, 1.1%). In the bivariate binary logistic regression analysis, being a blood donor older than 45 years in 2012 and employed was significantly associated with HCV infection. However, in the multivariable analysis, being employed and a blood donor in 2012 were the only significant factors associated with HCV infection. The odds of HCV among those who donated blood in 2012 were two times higher than among those who donated blood in 2010 (AOR = 2.09; 99%CI: 1.85, 5.09). Similarly, the odds of HCV among people who were employed donors were almost four times higher than among student donors (AOR = 4.35; 99%CI: 1.23, 15.23) (Table 4).

**Table 4:** Sero-prevalence of HCV infection according to socio-demographic characteristics of blood donors at the North Gondar District Blood Bank, northwest Ethiopia.

Variables	HCV		COR (99%CI)	AOR (99%CI)
	Reactive	Non-reactive		
**Age group**				
18–25	16	3,054	1.00	
26–35	22	2,169	1.94(0.83, 4.53)	
36–45	6	840	1.4(0.40, 4.70)	
>45	7	357	**3.74(1.15, 12.13)**	
**Sex**				
Male	44	5,267	1.00	
Female	7	1,153	0.73(0.25, 2.08)	
**Occupation**				
Student	5	1,837	1.00	1.00
Farmer	14	1,714	3.00 (0.78, 11.51)	3.12 (0.81, 12.00)
Employed*	27	2,284	**4.34 (1.23, 15.26)**	**4.35 (1.23, 15.23)**
Unemployed	5	585	3.13 (0.61, 16.08)	2.91 (0.56, 14.95)
**Year of donation**				
2010	12	1,994	1.00	1.00
2011	10	2,161	0.76 (.025, 2.32)	0.74 (0.86, 5.08)
2012	29	2,265	**2.13 (0.87, 5.17)**	**2.09 (1.85, 5.09)**

COR: Crude Odds Ratio; AOR: Adjusted Odds Ratio; CI: Confidence Interval; and

*Employed: government employed, private employed and self-employed

Type of donation was not included in the model because it has zero values in one of the cell.

### Sero-prevalence and associated factors of HIV infection

The HIV prevalence among blood donors was 145 (2.24%; 99%CI: 1.8%, 2.7%). HIV sero-prevalence was 2.3% in males and 0.71% in females. The age-specific distribution of HIV infection revealed that a high prevalence was detected among blood donors who were older than 45 years at 14 (3.9%). In the bivariate binary logistic regression analysis, age, occupation, and year of blood donation were significantly associated with HIV infection. However, in the multivariable analysis, only occupation was significantly associated HIV infections. Farmers (AOR = 4.02; 95%CI: 1.79, 9.03), employed (AOR = 3.75; 99%CI: 1.70, 8.28), and unemployed (AOR = 5.97; 99%CI: 2.43, 14.61) donors were more likely to be infected with HIV than student donors (Table 5).

**Table 5:** Sero-prevalence of HIV infection according to socio-demographic characteristics of blood donors at the North Gondar District Blood Bank, northwest Ethiopia.

Variables	HIV		COR (99%CI)	AOR (99%CI)
	Positive	Negative		
**Age group**				
18–25	46	3,024	1.00	
26–35	58	2,133	**1.79(1.07–3.00)**	
36–45	27	819	**2.17(1.2–4.10)**	
>45	14	350	**2.63(1.18–5.85)**	
**Sex**				
Male	123	5,188	1.00	
Female	22	1,138	0.82(0.45–1.50)	
**Occupation**				
Student	13	1,829	1.00	1.00
Farmer	48	1,680	**4.02 (1.79, 9.03)**	**4.02 (1.79, 9.03)**
Employed*	60	2,251	**3.75 (1.67, 8.28)**	**3.75 (1.70, 8.28)**
Unemployed	24	566	**5.96 (2.44, 14.61)**	**5.97 (2.43, 14.61)**
**Type of donation**				
Voluntary	4	378	1.00	
Replacement	141	5,948	2.24(0.6–8.33)	
**Year of donation**				
2010	36	1,970	1.00	
2011	46	2,125	1.18 (0.66, 2.11)	
2012	63	2,231	**1.55 (1.89, 2.66)**	

COR: Crude Odds Ratio; AOR: Adjusted Odds Ratio; CI: Confidence Interval; and

*Employed: government employed, private employed and self-employed

## Discussion

Blood transfusion is considered to be a potential risk factor for the transmission of viruses such as HBV, HCV and HIV, which are life-threatening and global public health problems. In this study, we found that the overall sero-prevalence of TTIs was 6.55% (99% CI: 5.76–7.34%), which is in agreement with a previous report from Hawassa, Ethiopia (7.0%) [16]. However, this prevalence is lower than other studies conducted in different part of Ethiopia, such as Gondar (9.5%) [15], Bahir Dar (43.2%) [18], Wolaita Sodo (29.5%) [17], and Jijiga (11.5%) [21]. The prevalence reported in this study was also lower compared to other African countries that reported an overall prevalence of viral infection ranging from 9.5% to 21.2% [30–32]. This may be because our study focused only on three viral infections (i.e., HIV, HBV, and HCV), whereas previous studies included these viral infections in addition to syphilis. Another possible reason for this low prevalence of TTIs in our study could be due to differences in time period because our study used data collected in 2010 to 2012. This is relatively recent when compared to data used by the majority of the above studies [15,18,30,31].

Our study revealed that the sero-prevalence of TTIs increased from 5.0% in 2010 to 9.28% in 2012. Although it does not demonstrate the trend of TTIs in the study area, as it is a three year retrospective data set, it may suggest that either the overall prevalence of TTIs is increased in the community overtime or the sensitivity of the test methods used to screen donated blood are improved as a result of changes in policies and strategies that governments have enforced to control TTIs. Another reason for this might be related to the shift of duty from the Red Cross Society to the National Blood Transfusion Service Agency in 2010 [29]. After 2010, the National Blood Transfusion Service Agency developed national blood policy, standards, operating procedures, and guidelines to ensure the safety and accessibility of blood. This involved advocating the importance of blood donation, recruiting blood donors via campaigns, and rigorous testing of donated blood for TTIs. This creates the opportunity to recruit more blood donors who have risky behavior for TTIs, which ultimately increases the magnitude of the problem. In support of this argument, our study revealed that the odds of HBV and HCV infections among people who donated blood in 2012 were almost two times higher compared to people who donated blood in 2010.

In our study, the sero-prevalence of HBV among blood donors was 3.6% (99%CI: 3.0%, 4.2%), which is lower compared to previous studies conducted in Ethiopia [15,16,18–21,33] and elsewhere in the world [30,34–37]. This lower prevalence might be attributed to differences in the specificity and sensitivity of the screening test. The blood bank in the Gondar district uses a conventional HBSAg test (i.e., the third generation ELISA test) for the screening of HBV. The conventional donor screening for HBsAg may yield serologically negative results despite the presence of HBV DNA [38]. Studies have shown that 20% of occult HBV infections are negative for all HBV seromarkers [39]. Therefore, if molecular techniques had been used for the screening, the magnitude most likely would have been more than the current finding. The other explanation might be a difference in the geographical variations that have been reported in the occurrence of viral infections, as well as a difference in the mode of prospective donor selection and study populations. However, when compared with the global prevalence of chronic HBV infection category, it is within the range of intermediate clusters (2–7%) [40], indicating that HBV is common in the study area.

The prevalence of HCV infection in this study was 0.8% (99%CI: 0.5%, 1.1%). Comparable figures were reported in Gondar, Ethiopia (0.7%) [15], Hawassa, Ethiopia (0.6%) [16], and Dessie, Ethiopia (0.61%) [33]. However, a higher prevalence was observed in previous studies carried out in Ethiopia [18–20] and elsewhere in the world [30,37,41–44]. On the other hand, a lower prevalence was observed in previous studies done in Jijiga, Ethiopia (0.4%) [21]. The possible explanation for the variation in the magnitude of HCV infections across studies might be due to differences in risk behaviors across different geographical locations and differences in socio-cultural practices. Cultural practices such as tattooing and sharing of contaminated materials, such as needles are common among uneducated people [45]. These practices could increase the risk of being infected with HCV. Employed donors were four times more likely to be infected with HCV when compared to student donors. This might be related to occupational injuries, such as needle sticks in healthcare settings [46,47].

Regarding the prevalence of HIV infection, our study demonstrated that 2.24% (99%CI: 1.8%, 2.7%) of blood donors were sero-reactive, which is similar to the result obtained from the Ethiopian Demographic and Health Survey Report [48]. The highest prevalence of HIV infection (4.1%) was observed in donors who were unemployed. Similar results have been reported in previous studies [15,49,50]. This might be due to low socio-economic levels of unemployed donors, as they are most likely to indulge in risky sexual relationships that may expose them to TTIs. Employed donors were also at higher risk of HIV-infection compared to student donors. The reason for high prevalence of TTIs among employed donors as compared to student donors might be related to increased exposure to TTIs, most likely due to the possibility of engaging in more risky behaviors over time and the transfusion of unsafe blood and/or blood products in their lifetime. Similarly, farmers were also at higher risk of having HIV infection than students. This might be related with the lower awareness of farmers about the mode of transmission and prevention of HIV. Previous studies conducted in Ethiopia showed that farmers had poor knowledge about HIV prevention methods, which may lead to unprotected sexual practices [51].

The overall prevalence of HIV, HBV and HCV co-infection in our study was 4 (0.062%). Different studies conducted in Ethiopia also report that the co-infection rate of these TTIs range from 0.19 to 4.8% [15,16,32]. The most common co-infection was HBV-HCV (75%). Comparable results were observed in Hawassa, Ethiopia (66.7%) [16], and Ghana (45.5%) [37]. The occurrence of co-infections could be because these infections share similar modes of transmission [34].

The highest prevalences of HBV (7.0%) and HIV (4.1%) were observed in those donors who were unemployed. This is supported by findings from other studies [15,49,50]. Similarly, unemployed donors were four times more likely to be infected with HBV and six times more likely to be infected with HIV than student donors. This might be due to low socio-economic levels of unemployed donors, as they are most likely to indulge in risky sexual relationships that may expose them to TTIs. Moreover, as a consequence of economic problems, unemployed donors may experience risky practices, such as sharing of personal care items like razors or toothbrushes, sharing of sharp kitchen materials, and having sexual contact with a person infected with TTIs. Furthermore, farmer donors were two times more likely to be infected with HBV and four times more likely to be infected by HIV compared to student donors. This might be due to risky cultural practices such as tattooing and sharing of sharp materials, which are common in uneducated people, including farmers [45]. Such practices could increase the risk of infection with transfusion-transmissible pathogens.

The limitation of this study is related to the retrospective nature of its design in that it did not include all risk factors associated with TTIs. With this limitation, the study attempted to demonstrate the sero-prevalence of the major TTIs among blood donors in the North Gondar District.

### Conclusion

The prevalence of TTIs is substantial and has increased over time. Being a farmer, unemployed and employed were found to be significantly associated with TTIs. Hence, more vigilance in routine screening of donated blood prior to transfusion is needed. Further community-based studies to identify societal risk factors for blood-borne pathogens are necessary.

## Supporting information

S1 Dataset “S1 Dataset used for analysis” includes socio-demographic characteristics, transfusion-transmissible viral infections, donor type and year of donation (.sav). (SAV)

## Abbreviations

ELISA: Enzyme Linked Immunosorbent Assay
HBV: Hepatitis B Virus
HBsAg: Hepatitis B Surface Antigen
HCV: Hepatitis C Virus
HIV: Human Immunodeficiency Virus
TTI: Transfusion transmitted infection
WHO: World Health Organization
